# Impact of the Sensory Neurons on Melanoma Growth *In Vivo*

**DOI:** 10.1371/journal.pone.0156095

**Published:** 2016-05-26

**Authors:** Anton A. Keskinov, Victor Tapias, Simon C. Watkins, Yang Ma, Michael R. Shurin, Galina V. Shurin

**Affiliations:** 1 Department of Pathology, University of Pittsburgh Medical Center, Pittsburgh, Pennsylvania,United States of America; 2 Department of Neurology, University of Alabama at Birmingham, Birmingham, AL, United States of America; 3 Department of Cell Biology and Physiology, University of Pittsburgh Medical Center, Pittsburgh, Pennsylvania, United States of America; 4 Department of Immunology, University of Pittsburgh Medical Center, Pittsburgh, Pennsylvania,United States of America; Rutgers University, UNITED STATES

## Abstract

Nerve endings are often identified within solid tumors, but their impact on the tumor growth and progression remains poorly understood. Emerging data suggests that the central nervous system may affect cancer development and spreading via the hypothalamic-pituitary-adrenal axis and autonomous nervous system. However, the role of the afferent sensory neurons in tumor growth is unclear, except some reports on perineural invasion in prostate and pancreatic cancer and cancer-related pain syndrome. Here, we provide the results of primary testing of the concept that the interaction between melanoma cells and sensory neurons may induce the formation of tumor-supporting microenvironment via attraction of immune regulatory cells by the tumor-activated dorsal root ganglion (DRG) neurons. We report that despite DRG cells not directly up-regulating proliferation of melanoma cells *in vitro*, presence of DRG neurons allows tumors to grow significantly faster *in vivo*. This effect has been associated with increased production of chemokines by tumor-activated DRG neurons and attraction of myeloid-derived suppressor cells both *in vitro* and *in vivo*. These initial proof-of-concept results justify further investigations of the sensory (afferent) nervous system in the context of tumorigenesis and the local protumorigenic immunoenvironment.

## Introduction

Emerging data from the pre-clinical and clinical studies indicate a distinctive role of the nervous system in tumor growth and progression. Psychosocial studies reveal alterations in the brain activity in patients with solid tumors and the analysis of neuromediators and neuroendocrine hormones demonstrates their important role in the oncogenic process [1–3]. However, beyond a well-established concept of the Central Nervous System (CNS) as a regulator of tumor growth via the hypothalamic-pituitary-adrenal axis [4], much less is known about the role of the Peripheral Nervous System (PNS) in this process. The studies of the nervous system role in cancer mainly focus on the Autonomic (efferent) branch of the PNS. Both sympathetic and parasympathetic divisions of the Autonomic Nervous System regulate tumor cell growth, migration, and invasiveness since catecholamines can bind to α- and β-adrenergic receptors while acetylcholine can bind to nicotinic and muscarinic receptors which are expressed on tumor cells and stromal elements within the tumor microenvironment [5, 6]. Moreover, a significant decrease in tumor growth after sympathectomy in experimental models has been reported [7–9], while higher incidences of cancer have been observed both in parasympathetic denervation models and in patients who underwent vagotomy [10, 11].

Notably, in addition to the efferent Autonomic Division, the PNS also includes the sensory (afferent) division. Neurons of the sensory nervous system send information to the CNS from internal organs and external stimuli. Their primary functions include noci-, proprio-, mechano- and thermoception which dictate the abundance of their sensory terminals within the body. At most the cell soma of primary afferent neurons localizes in the dorsal root ganglion (DRG) within the vertebral column. Interestingly, DRG neurons are not protected by Blood-Brain or Blood-Nervous Barrier, which makes them a rare non-immune-privileged type of neurons within the body [12]. In addition to neurotrophic factor receptors, DRG neuronal cells also express different chemokine receptors, Toll-like receptors and Tumor necrosis factor receptors [13, 14]. They are capable of releasing neuropeptide transmitters such as substance P, calcitonin gene-related peptide, vasoactive intestinal polypeptide, endothelin, as well as histamine and glutamate [15, 16].

The role of DRG neurons in cancer has been studied exclusively in the context of perineural tumor invasion and cancer-related pain syndrome [17, 18]. Observations confirming chemoattraction of tumor cells to DRG neurons along with immunohistochemistry staining of tumor biopsies suggest a possible role for the PNS in cancer. However, neither direct *in vivo* results nor potential mechanisms of tumor-regulating activity of the sensory nervous system are available. Here, we introduce a novel concept of the actual role of the sensory nervous system in the tumor growth process. A broad network of free nerve endings is abundantly spread within various tissues, especially in the skin. It may become a part of the first line of defense against invading tumor cells. On the other hand, tumor cells may exploit for their benefit the original “wound-healing” mechanism pre-programmed within the sensory afferents. We have speculated that the interaction between malignant cells and sensory neurons may induce the formation of tumor-supporting microenvironment via attraction of immune regulatory cells by tumor-activated DRG neurons.

The goal of this study has been to determine both direct and indirect effects of DRG neurons on tumor cell growth *in vitro* and *in vivo*. We have revealed that although DRG cells do not immediately up-regulate proliferation of melanoma cells *in vitro*, their presence significantly increases tumor growth *in vivo*. The contact between DRG and tumor cells results in an increased production of chemokines by tumor-activated DRG neurons, and as a result in migration of myeloid-derived suppressor cells (MDSC), which are known to support the establishment of the immunosuppressive and protumorigenic microenvironment [19]. MDSC attraction by DRG has been confirmed *in vitro* and *in vivo* in tumor-bearing mice. Therefore, our data demonstrate a significant impact of DRG cells on tumorigenesis associated with MDSC recruitment to the tumor site. These results provide a rationale for a further investigation of the sensory (afferent) nervous system in the context of tumorigenesis.

## Materials and Methods

### Mice

Pathogen-free C57BL/6 mice (7-8-week old) from Jackson Labs (Jackson Lab, Bar Harbor, Maine) were housed in a pathogen-free facility under controlled temperature, humidity, and 12-h light/dark cycle with a commercial rodent diet and water available *ad libitum*. Physical condition of the animals was monitored on a daily basis.All animal were euthanized according to the approved procedure from the most recent AVMA Guidelines on Euthanasia in the chamber by introducing to 100% carbon dioxide. A fill rate of 20% of the chamber volume per minute with carbon dioxide, added to the existing air in the chamber, was used. After removal from the chamber, cervical dislocation was applied. All studies were conducted in accordance with the National Institutes of Health guidelines for the Care and Use of Laboratory Animals and approved by the Institutional Animal Care and Use Committee of the University of Pittsburgh.

### Tumor cell cultures

Murine B16 melanoma cell line (ATCC) was maintained in a complete RPMI 1640 medium (GIBCO BRL) supplemented with non-essential amino acids, 10% heat-inactivated FBS (Gemini Bio-Products), 2 mM L-glutamine, 100 IU/ml penicillin and 100 μg/ml streptomycin (Invitrogen Life Technologies Inc.) When the cells were at subconfluence, they were harvested, washed and 1x10^6^ cells were cultured in 20 ml of complete PRMI 1640 medium with 10% FCS for 24 h. Then the medium was replaced with complete PRMI 1640 medium supplemented with 2% FCS. Forty-eight-hour condition medium was collected and centrifuged at 700*g* at RT for 5 min to pellet cells. The supernatant was collected and centrifuged at 2000*g* at 4°C for 10 min to remove cell debris.

### Dorsal Root Ganglion cell cultures

Mouse DRG neurons were isolated as described previously [20, 21] with small modifications. Cervical, thoracic and lumbar spinal regions were exposed, the roof of the vertebral canal was removed, and DRG were collected. Ganglia were digested consecutively with papain and collagenase type 2/neutral protease solutions to obtain a single DRG cell suspension. Papain and Collagenase type II (CLS2) were from Worthington; Dispase type II was from Roche. DRG cell cultures were plated on round glass coverslips (EMS) coated with Poly-D-Lysine (Sigma) and Laminin (Sigma). The serum-free DRG medium consisted of Neurobasal A (Gibco), B-27 Supplement (Gibco), 2mM L-Glutamine-Pen-Strep (Gemini) and 2mM GlutaMAX Supplement (Gibco).

### Immunocytochemistry, Cell Imaging and Analysis

DRG cells on coverslips were fixed with 4% paraformaldehyde in PBS (pH 7.5) for 20 min at RT and rinsed 3 times in PBS for 10 min intervals. Non-specific binding of secondary antibody was blocked with 5% BSA in PBS (blocking buffer) for 45 min at RT. Cell cultures were incubated with mouse anti-tubulin β3 (TUBB3) (1:1000, Biolegend) primary antibody overnight at 4°C, rinsed three times for 10 min at RT with PBS and then incubated with donkey anti-mouse IgG conjugated to CY3 (1:1000, Jackson Immuno) in blocking buffer for 2 h at RT. Cultures were rinsed again with PBS and H33342 dye (1:3000, Sigma-Aldrich) was added for 3 min at RT for visualization of the nuclei.

Wide-field epifluorescence was used for DRG cell imaging. Images were collected using a Nikon Eclipse 90i upright fluorescence microscope equipped with five fluorescent channels and high N.A. plan fluor/apochromat objectives had been used for DRG cell imaging[22]. Images were collected using Nikon NIS-Elements software and Q-imaging CCD camera (QImaging; Retiga EXi Fast 1394). The stage was scanned using a Renishaw linear encoded microscope stage (Prior Electronics). All slides were scanned under the same conditions for magnification, exposure time, lamp intensity and camera gain. Quantitative analysis was performed on fluorescent images generated in 2 fluorescent colors (stained for B3T and H33342) using the Nikon NIS-Elements software. The entire region of the coverslip was delineated as an active region of interest (ROI) (excluding the edges to eliminate some cell aggregation and fluorescence saturation) and used for analysis (~75% of the total area). Quantification and 3D remodeling of images was carried out by using the FilamentTracer module of Imaris (Bitplane). The Cy3 (B3T) channel was utilized to evaluate DRG neurite length, area, volume, and the number of segments / branches per slide. Systematic ROI delineation, using a sampling grid (of 8 squares), which basically comprises the entire image of the slide, was utilized for an unbiased neurite examination.

### Analysis of Cytokine production

For the generation of DRG supernatants from control and tumor-treated cultures, DRG were cultured for five days, and then the medium was changed with fresh DRG medium containing either 10% (v/v) B16 melanoma-conditioned medium or 10% (v/v) RPMI 1640 complete medium (control). After 48 h, the medium was substituted with fresh DRG medium and two days later supernatants were collected for cytokine determination by Proteome Profiler Array ARY006 (R&D Systems). Samples were obtained from four mice/group. Each sample was diluted and mixed with a cocktail of biotinylated detection antibodies and then incubated with Mouse Cytokine Array membrane. Therefore, any cytokine/detection antibody complex being present had been bound by its cognate immobilized capture antibody on the membrane. After washing, Streptavidin-Horseradish Peroxidase and chemiluminescent detection reagents were added sequentially. Array images were obtained from X-ray films after 20 min exposure time and analyzed by densitometry for Integral optical density (IOD) using UN-SCAN-IT gel Software package (Ver.7.1, Silk Scientific Inc.). The IOD of each cytokine was normalized to the corresponding positive control IOD results. Production of the following proteins by DRG cells was assessed: CXCL13, C5a, G-CSF, GM-CSF, CCL1, CCL11, sICAM-1, IFN-γ, IL-1α, IL-1β, IL-1ra, IL-2, IL-3, IL-4, IL-5, IL-6, IL-7, IL-10, IL-13, IL-12p70, IL-16, IL-17, IL-23, IL-27, IP-10, CXCL11, KC, M-CSF, CCL2, CCL12, CXCL9, CCL3, CCL4, CXCL2, CCL5, CXCL12, CCL17, TIMP-1, TNF-α and TREM-1.

### Tumor cell proliferation assay

The impact of DRG cells on B16 cell proliferation was assessed using the ^3^H-thymidine incorporation assay. B16 cells were co-cultured with medium, DRG neurons or DRG conditioned medium for 72 h, harvested, washed and seeded as 2.5x10^3^ cells per well/200 μl complete medium in 96-well plates for 48 h. Cells were then pulsed for 16–18 h with ^3^H-thymidine (1 μCi/well, 5 Ci/mmol; DuPont-NEN) and harvested onto GF/C glass fiber filters (Whatman Intl. Ltd) using MACH III Microwell Harvester (Tomtec). ^3^H-thymidine incorporation was determined on MicroBeta TRILUX liquid scintillation counter (WALLAC) and expressed as count per minute (cpm).

### Analysis of Tumor Cell Cycle

The effect of DRG cells on melanoma cell proliferation was additionally determined using Cayman Cell Cycle Phase Determination Kit. Briefly, B16 cells were co-cultured with medium, DRG cells or DRG conditioned medium for 72 h, harvested, washed, fixed and staining with Propidium Iodide and Staining Solution. Cells were analyzed on FACScan (Becton Dickinson) using FlowJo software package.

### Isolation of MDSC for in vitro experiments

MDSC were isolated from the bone marrow samples using CD11b^+^ Myeloid-Derived Suppressor Cell Isolation Kit (Miltenyi Biotec) according to the manufacturer’s protocol. Briefly, bone marrow cells were depleted of erythrocytes and treated with Ly-6G-Biotin and Biotin microbeads. Cell suspensions were then loaded in the column to select Ly-6G^+^ cells. Harvested Gr-1^dim^Ly-6G^-^ cells were labeled with anti-GR-1-Biotin and Streptavidin Microbeads, and positively labeled cells were isolated. The enriched population of MDSC (9x10^6^ cells/ml) was re-suspended in RPMI 1640 complete medium, cultured for 24 h and used in other experiments.

### MDSC migration assay

Control and tumor-treated DRG conditioned media were added into 24 well plates (600 μl per well) and 2.5x10^5^ MDSC in 100 μl of DRG medium were set over in cell inserts with 5 μm pore size (Corning). After a 4-h incubation at 37°C, the Transwell inserts were removed, and cells from the lower chamber were collected. Cells transmigrated through the membrane were acquired on FACScan (BD Biosciences) for 60 sec. Data reported as the mean number of transmigrated cells from triplicate wells as described earlier [23].

### Animal tumor models and experimental design

For evaluation of the effect of DRG cells on tumor growth *in vivo*, in the first set of studies, animals were injected s.c. with a mixture of B16 cells (5x10^4^cells) and DRG cells (1x10^4^cells) in 100μl PBS. Administration of melanoma and DRG cells separately served as a control. Tumor size was estimated 2–3 times per week and expressed as mm^2^ area.

In the second set of studies, animals were initially injected with DRG cells (1x10^4^ cells) in the Matrigel (500μl ECM gel (Sigma) diluted 1:1 in RPMI 1640). Two weeks later, B16 cells (5x10^4^/300μl PBS) were administered into the Matrigel plaques. Animals were sacrificed two weeks post tumor inoculation, Matrigel plaques were weighted, and single cell suspensions from tumor specimens were prepared using Cell Recovery Solution (Corning, USA) and Tumor Dissociation Kit (Miltenyi Biotec). After tumor digestion, cells were washed, labeled with anti-CD11b, Ly6G, Ly6C and CD45 antibodies (Biolegend Inc.) directly conjugated to FITC, PE/Cy7, APC and PerCP-CY 5.5 and analyzed by flow cytometry (Becton Dickinson). MDSC levels in the Matrigel plaques were assessed as the percentage of total cells and CD45+ cells in the tested tissues and compared with control Matrigel plaques without DRG cells. All animal experiments included 6–7 mice per group and were repeated at least 2–3 times.

For the IHC analysis of tumor tissues, specimens were fixed in ice-cold methanol for 20 min at -20°C and rinsed with PBS, followed by permeabilization by 3% H_2_O_2_ in methanol. Non-specific binding of secondary antibody was blocked with 3% BSA in PBS (blocking buffer) for 45 min at RT. Slides were stained with rabbit anti-mouse Neurofilament H primary antibody (1:200 dilution with 3% BSA, 60 min, RT, Chemicon), followed by biotinylated goat anti-rabbit IgG secondary antibody (1:500 dilution with 3% BSA, 60 min, RT, Vector Laboratories). After three washes in PBS, slides were incubated with RTU ABC reagent (Vector Laboratories) for 30 min, then rinsed with PBS and incubated with DAB solution (BD Biosciences 550880) for 30 sec and counterstained with hematoxylin (Vector Laboratories) for 25 sec. Slides were reviewed on an Olympus BX45 microscope with UPlanFLN 10x/30 objective at RT and images were captured using a Spot Insite 2Mp CCD camera and Spot software v.4.6.

### Statistical analysis

For a single comparison of two groups, the Students *t*-test was used after evaluation of normality. If data distribution was not normal, a Mann-Whitney rank sum test was performed. For the comparison of multiple groups, analysis of variance was applied. SigmaStat Software was used for data analysis (SyStat Software, Inc.). For all statistical analyzes, *p*<0.05 was considered significant. All experiments were repeated at least two times. Data are presented as the mean ± SEM.

## Results

### DRG neurons accelerated melanoma growth in vivo

**Fig 1:**
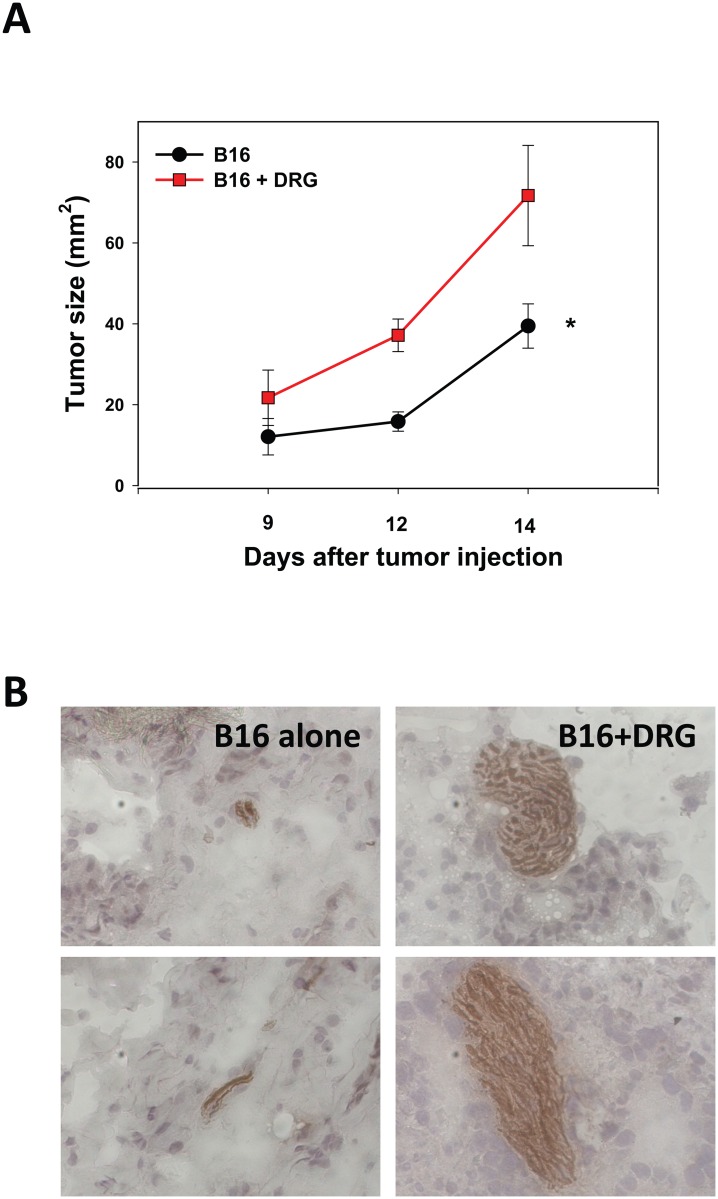
Co-administration of DRG neurons with B16 melanoma cells up-regulated tumor growth in mice. C57BL/6 mice (5 mice/group) received s.c. injection of 50x10^3^ tumor cells alone or mixed with cultured DRG neurons (1:5 cell:cell ratio). Tumor size was assessed using a caliper and expressed in mm^2^ (A). Administration of DRG cells alone, as expected, did not induce any tumor growth. Tumor tissues were harvested two weeks later, and the presence of live DRG neurons in the tumor was determined by DAB staining after 14 days using an antibody against the anti-Neurofilament H epitope (B). Left panels show control tumors (administration of B16 cell alone), right panels show tumors growing in mice receiving B16+DRG neurons injections. Tumor size is expressed as the mean ± SEM. *, p<0.05 (ANOVA, n = 3).

First, we tested whether DRG neurons affected melanoma growth by comparing tumor growth in mice receiving B16 cells alone and B16 cells mixed with cultured DRG neurons. The results revealed that co-administration of B16 and DRG cells resulted in a significant up to two-fold (p<0.05, ANOVA) acceleration of melanoma growth in immunocompetent mice (Fig 1A). Importantly, IHC analysis of tumor tissues harvested two weeks after cell inoculation confirmed the survival of DRG neuron in the tumor mass in mice receiving B16+DRG cells injections (Fig 1B). Thus, these data suggest that DRG neurons were capable of increasing melanoma growth *in vivo*.

**Fig 2:**
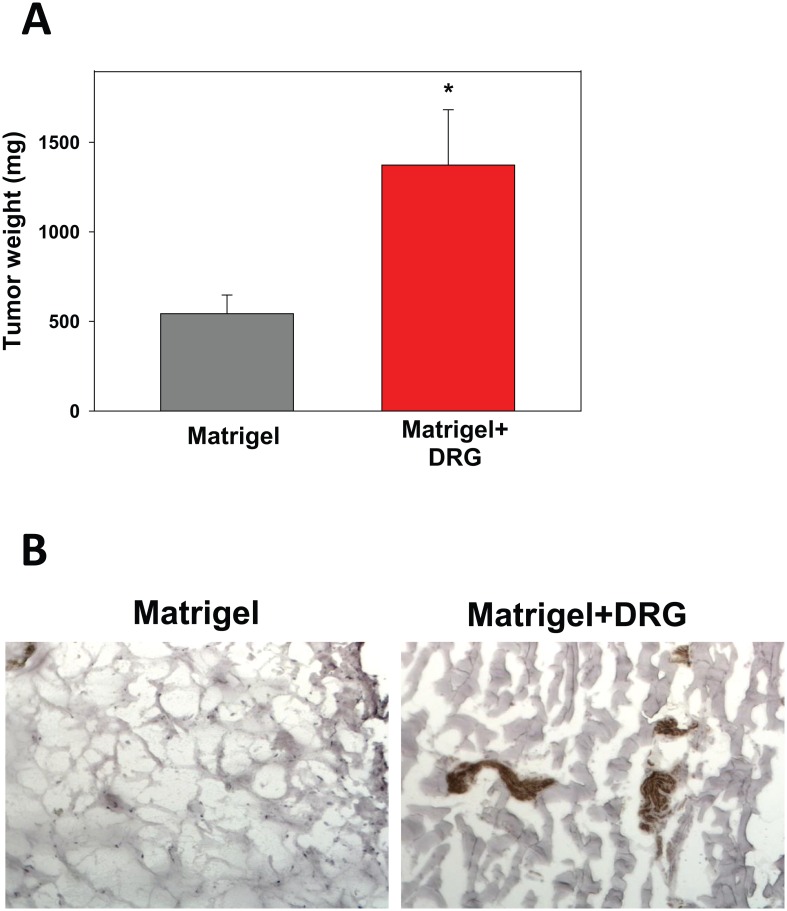
Pre-existence of DRG neurons within the Matrigel increased B16 melanoma growth in the Matrigel in mice. C57BL/6 mice (5 mice/group) received s.c. injection of control Matrigel (500μl ECM gel diluted 1:1 in RPMI 1640) and Matrigel with cultured DRG neurons (1x10^4^ cells). B16 cells (5x10^4^/300μl PBS) were administered into the Matrigel plaques two weeks later. Animals were sacrificed two weeks post tumor inoculation, and Matrigel plaques were weighted to characterize tumor growth (A). 50x10^3^ tumor cells alone or mixed with cultured DRG neurons (1:5 cell:cell ratio). (A). The presence of live DRG neurons in the Matrigel harvested two weeks after administration, i.e., right before the tumor cell injection, was determined by immunohistochemistry with anti-Neurofilament H antibody as described in Materials and Methods (B). Left panel show control Matrigel, right panel show Matrigel with added DRG neurons two weeks after injections. Matrigel weight is expressed as the mean ± SEM. *, p<0.05 (ANOVA, n = 3).

To confirm these results and to test the hypothesis that the pre-existence of sensory neurons in the tissue may stimulate tumor growth *in vivo*, melanoma cells were *in vivo* inoculated into either the empty Matrigel plaque or Matrigel plaque containing DRG cells established for two weeks beforehand. Fig 2A shows that melanoma cells grew significantly faster (p<0.05) in the Matrigels with pre-existing DRG cells. Immunohistochemical analysis of neurons using neurofilament H staining confirmed viability of DRG neurons within the Matrigel two weeks after Matrigel+DRG cells administration (Fig 2B). Altogether, these results suggest that interaction between melanoma cells and DRG neurons resulted in accelerated tumor growth *in vivo*. Thus it raised the question whether DRG neurons could directly up-regulate proliferation of B16 melanoma cells.

### DRG neurons do not accelerate proliferation of B16 melanoma cells in vitro

**Fig 3:**
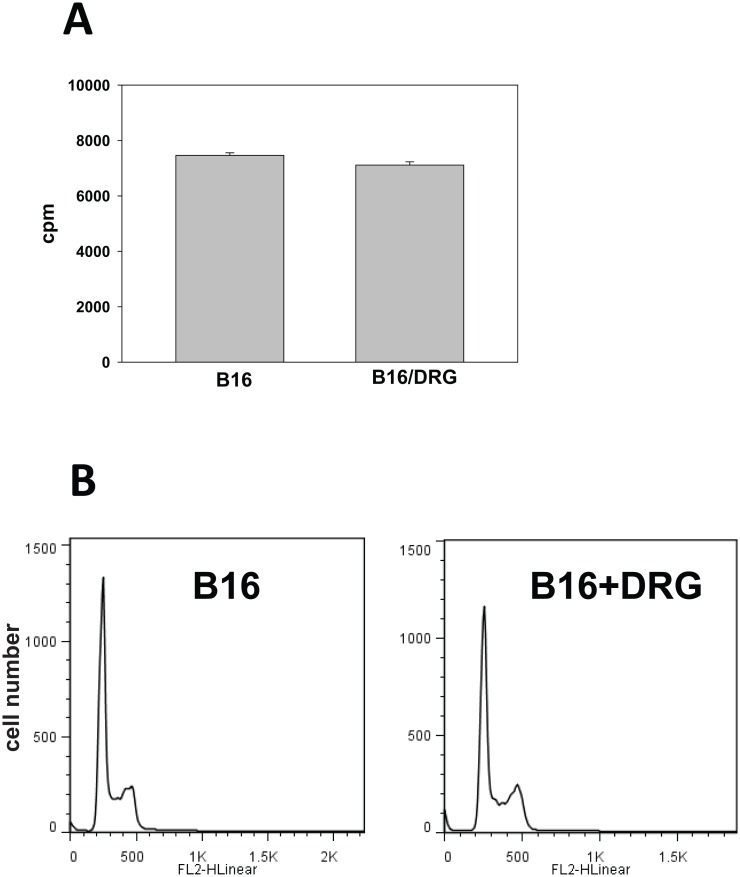
DRG neurons do not affect proliferation of B16 cells in vitro. B16 cells were cultured in the presence of DRG culture medium (10% v/v), and DRG conditioned medium (10% v/v) and cell proliferation were determined by the ^3^H-thymidine incorporation assay as described in Materials and Methods (A). The results are expressed as counts per minute (cpm) and shown as the mean±SEM (n = 3). Analysis of cell cycle was done by flow cytometry using propidium iodide (PI) to label DNA content in B16 cells cultured with and w/o DRG conditioned medium (B). The results of a representative experiment are shown (n = 2).

Up-regulation of melanoma growth by DRG neurons *in vivo* might be explained by a direct stimulation of melanoma cell proliferation by neurons. To test this possibility, the proliferation of B16 cells was determined with and w/o addition of the DRG-conditioned medium (10% v/v). DRG medium added to B16 cell cultures served as a control. Fig 3A shows that proliferation of tumor cells, assessed by 3H-thymidine incorporation, had not been markedly altered after addition of DRG conditioned medium: 7461±94 cpm versus 7112±116 cpm in control cultures (p>0.1, n = 3). These results were confirmed in the additional experiments where Cell Cycle Phase was determined: G0/G1: 57.3±2.5%, S: 12.4±2.1% and G2/M: 21.3±3.3% in B16+DRG cultures versus G0/G1: 59.2±1.9%, S: 10.4±1.8% and G2/M: 20.6±2.3% in control B16 cultures (p>0.1, n = 2) (Fig 3B). Thus, these results suggest that DRG neuron-derived factors did not directly up-regulate proliferation of B16 cells. Therefore, the direct effect of DRG cells on melanoma cells cannot explain the accelerated growth of tumor in the presence of DRG *in vivo*, as shown in Figs 1and 2. However, it is possible that DRG neurons can be activated by tumor cells to produce factors that indirectly modulate tumor growth *in vivo*.

### B16 melanoma cells activated DRG neurons resulting in upregulation of chemokine expression

**Fig 4:**
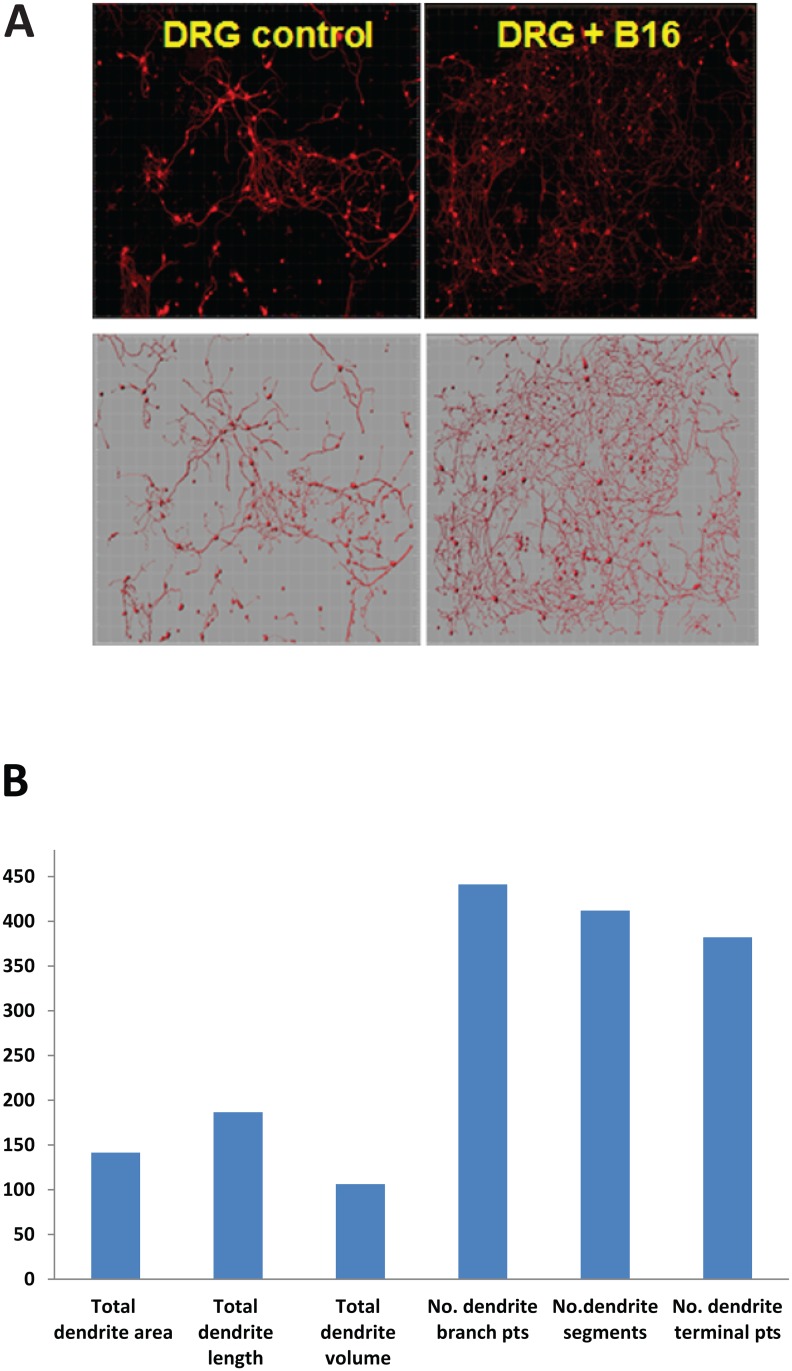
B16 cells stimulated the growth of DRG neurons *in vitro*. DRG cultures were treated with B16-conditioned medium (10% v/v, 72 h), stained for Neuronal class III ß-tubulin (A, upper panels) and subjected to 3D neuron and dendrite reconstruction (A, lower panels).Quantitative analysis was performed to determine DRGdendritic length, area, volume, and the number of segments and t branches per slide using the FilamentTracer module of Imaris (Bitplane) software package as described in Materials and Methods (B). Bars represent morphometric parameters of neurons treated with B16 conditioned medium as the percentage of control non-treated neurons. Representative images and automated data from one experiment are shown. Data from four independent cultures were combined to determine means±SEM.

Next, to test the hypothesis that B16 cells might affect DRG neurons, DRG cultures were treated with B16 conditioned medium (10% v/v) for 72 h and subsequent cell morphometric examination was carried out. As shown in Fig 4A, B16-derived factors visually altered cultured neurons stained with Neuronal Class III ß-Tubulin antibody (upper panels), which had been confirmed by the mathematical remodeling of obtained images (lower panels). Morphometric analysis of treated neuronal cultures revealed a significant increase in morphological parameters of neuronal cells, including dendrite branches and dendrite segments (up to 4-fold), total dendrite length (up to 2-fold) and total dendrite volume (up to 1.5-fold) (Fig 4B). These results suggest that DRG neurons can be activated by melanoma cells and rise the question about the biological significance of tumor-induced activation of neurons.

**Fig 5:**
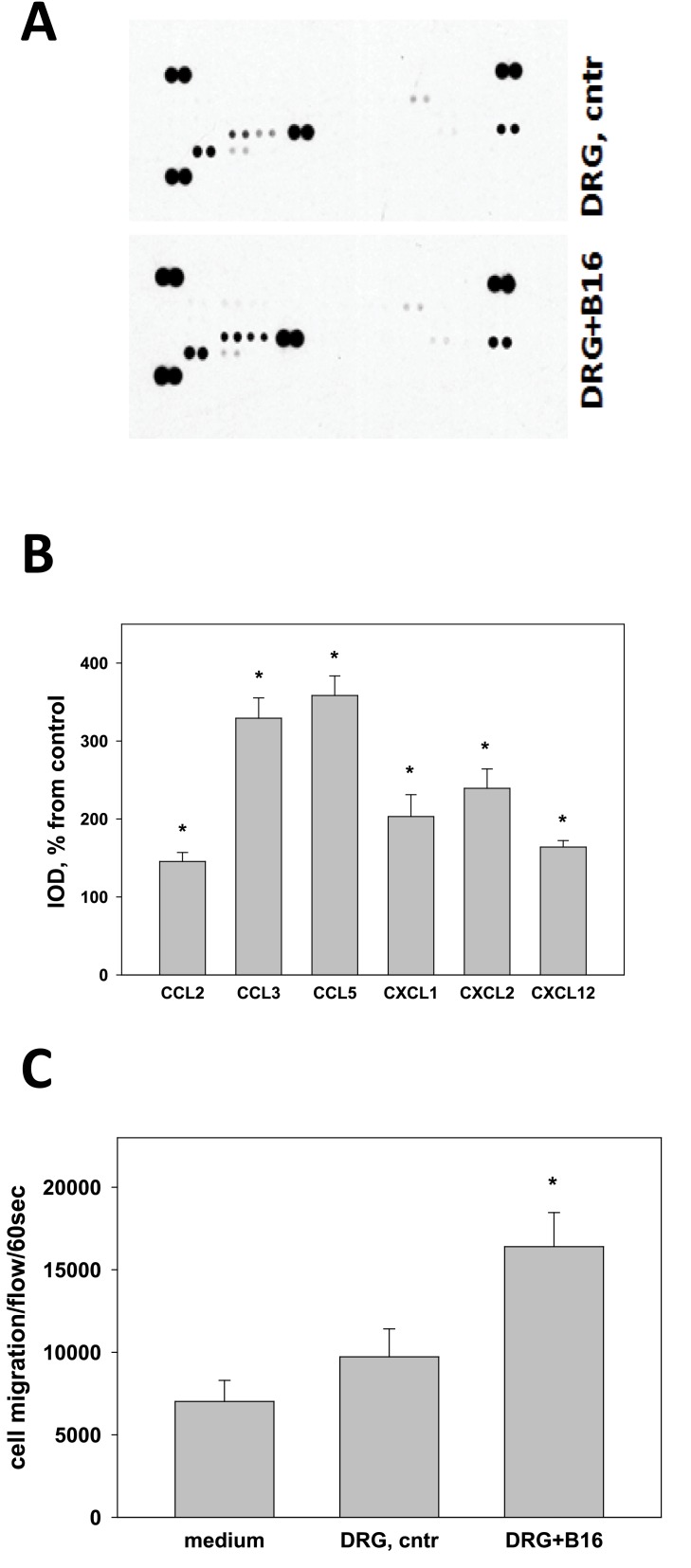
B16 cells upregulate expression of MDSC chemokines in DRG neurons and chemoattraction of MDSC *in vitro*. DRG were cultured for five days; then the medium was replaced with fresh DRG medium containing either 10% (v/v) B16 melanoma-conditioned medium or 10% (v/v) RPMI 1640 complete medium (control). Two days later, the medium was substituted with fresh DRG medium and in 48 h supernatants were collected for cytokine determination using the Proteome Profiler Mouse Array Kit (A). Array images were analyzed by densitometry for Integral optical density (IOD) using UN-SCAN-IT gel software package. The IOD of each cytokine was normalized to the corresponding positive control IOD results (B). Bars represent the fold increase in expression of selected chemokines and are shown as the mean±SEM. *, p<0.05 (Student *t*-test, n = 4). Migration of bone marrow-derived MDSC to control and B16-treated DRG conditioned media (as in A) was determined by a chemotaxis assay as described in Materials and Methods (C). Results are shown as the mean±SEM. *, p<0.05 (ANOVA, n = 3).

Next, we tested whether melanoma cells may alter production of common cytokines, chemokines and growth factors by DRG neurons using the Proteome Profiler Mouse Array kit and revealed a significant up-regulation of expression of chemokines that attract myeloid-derived suppressor cells, including CCL2, CCL3, CCL5, CXCL1, CXCL2 and CXCL12 (Fig 5). For instance, expression of CCL3, CCL5 and CXCL2 in melanoma-treated DRG cells increased up to 3.5-, 4- and 3-fold, respectively, compared to control DRG cells (p<0.05, n = 3, Fig 5B). To verify the biological significance of these results, we compared attraction of MDSC to control and B16-treated DRG neurons in the chemotaxis assay. The results demonstrated that chemoattraction of MDSC to melanoma-treated DRG neurons was significantly higher than attraction to control DRG cultures (p<0.05, n = 3, Fig 5C). Thus, these results suggest that melanoma cells can activate DRG neurons and increase expression of chemokines that attract MDSC *in vitro*.

### Melanoma growth in the presence of DRG neurons was associated with increased levels of tumor-infiltrating MDSC in vivo

**Fig 6:**
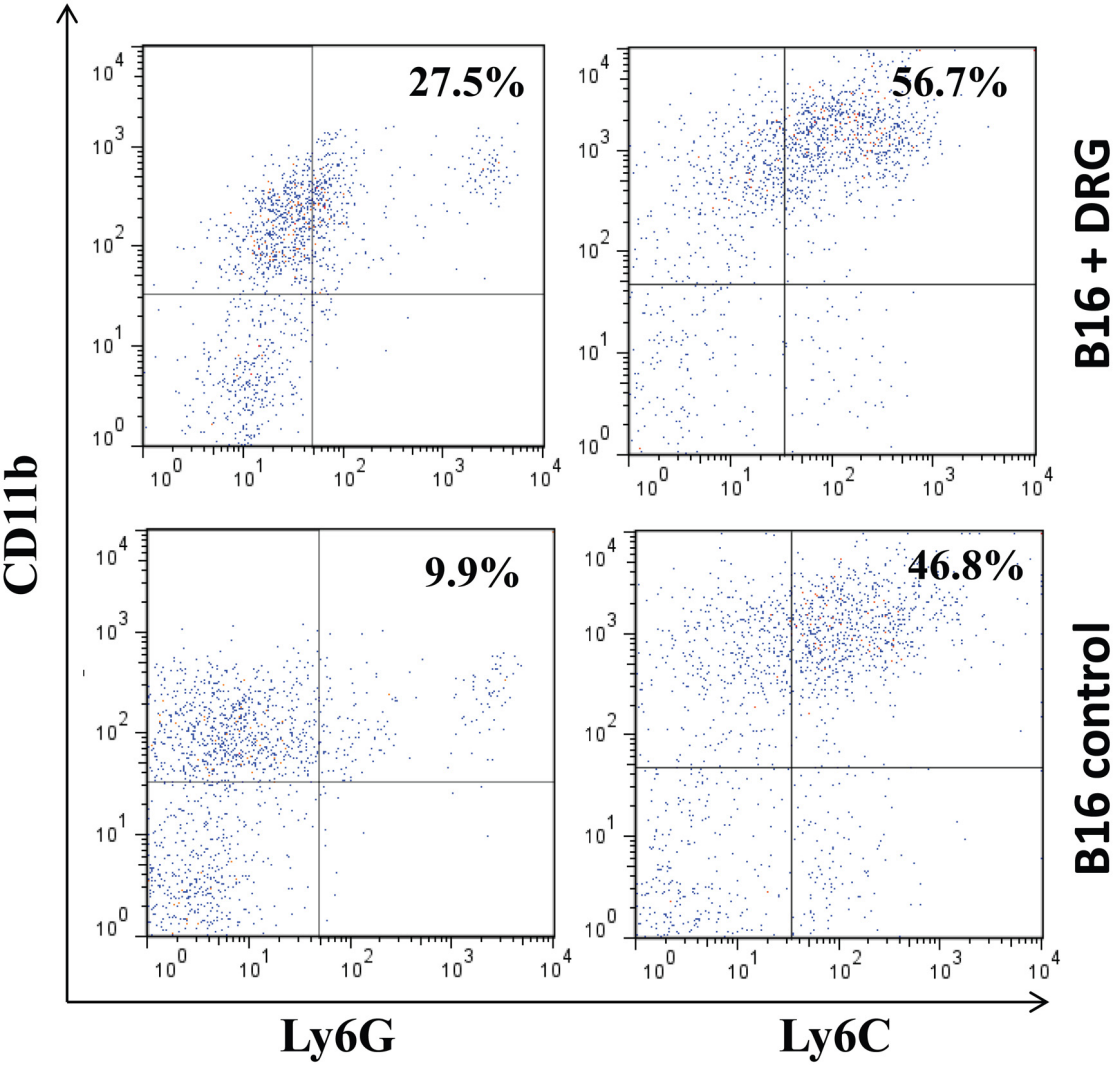
Accelerated growth of melanoma in the presence of DRG neurons was accompanied by an increased tumor infiltration by MDSC *in vivo*. C57BL/6 mice received s.c. injection of control Matrigel and Matrigel with DRG neurons as described in Fig 2 legend. B16 cells were administered into the Matrigel plaques two weeks later. Animals were sacrificed two weeks post tumor inoculation, and Matrigel plaques were harvested, dissolved and subject for the presence of granulocytic CD11b^+^Ly6G^+^ and monocytic CD11b^+^Ly6C^+^ MDSC among CD45^+^ leukocytes by flow cytometry. The results of a representative experiment from six mice analyzed in two independent studies are shown.

Finally, we tested whether the level of MDSC in melanoma mass growing in the presence of DRG neurons was higher than in control tumors. Matrigel plaques with B16 cells growing either alone or in the presence of pre-established DRG neurons (as in Fig 2) had been dissolved and infiltrating CD11b^+^GR-1^+^ MDSC were assessed among CD45^+^ leukocytes by flow cytometry (Fig 6). It unveiled a significant up to 3-fold increase in granulocytic CD11b^+^Ly6G^+^ MDSC and less prominent increase in monocytic CD11b^+^Ly6C^+^ MDSC in tumors developed with DRG cells in comparison with control tumors: 25.3±3.8% and 54.5±6.8% versus 7.8±1.2% and 44.1±5.4%, respectively. These results show that accelerated growth of melanoma in the presence of DRG cells *in vivo* had been accompanied by an augmented attraction and homing of MDSC.

In summary, our data demonstrated that the presence of DRG neurons accelerated melanoma growth *in vivo*. This process could be attributed to the activation of DRG neurons by malignant cells and release of chemoattractants for MDSC at the tumor site; meanwhile, the presence of MDSC created the specific tumor microenvironment to support tumor growth and progression.

## Discussion

Potential modulatory properties of the peripheral nervous system in several aspects of tumorigenesis have been investigated. The initial straightforward approach has relied on the assessment of the nerve fibers within the tumor foci. However, most data from published observations remain highly controversial. While some researchers indicate the presence and prognostic significance of the intratumoral nerve fibers [9, 17, 24–27], the other report reveal the nerve bundles only within surroundings of the tumor, and data denote absence of any nerve fibers within the tumor lesions [28–31]. Possible explanations for this contradiction may include the variability of stages and types of cancer tested for nerve fibers, divergent specificity of utilized antibodies, and limited applicability of thin tissue slides for the analysis of nerve fiber’s presence. For instance, anti-PGP 9.5 antibody, the most commonly used staining agent in these studies, does not seem to be the discrete marker for the nerve fibers within the tumor tissue [32]. Furthermore, the majority of immunohistochemical tests for tumor innervation have had no intention to distinguish between the autonomic (sympathetic and parasympathetic) and sensory (afferent) nerve fibers within the tumor tissue. The latter becomes a significantly limiting factor for obtained data to be used as a key to understanding the role of PNS in cancerogenesis.

The alternative approach has been used by *in vitro* studies of the sensory DRG neurons and their interactions with tumor cell lines. Several studies have demonstrated a bidirectional effect: an increase of both the migratory potential of tumor cells and the neurites extension by neurons [33]. This observation has been reported for human prostate cancer cells [17, 33], pancreatic cancer cell lines [18, 34–36] and colon adenocarcinoma cells [27]. Contrarily, some data demonstrate the class 3 semaphorin-mediated inhibition of axon outgrowth in DRG cell cultures by colon and melanoma cell lines [37]. Here, we have shown that melanoma cells activate DRG neurons *in vitro* and revealed an up-regulated expression of several chemokines in tumor-treated DRG neurons (Figs 2 and 3). Interestingly, our new data suggest that DRG cells isolated from melanoma-bearing mice also produce higher levels of certain chemokines when compared with DRG cells harvested from tumor-free mice (Keskinov et al., unpublished). These interesting findings suggest that the effect of growing tumor on PNS neurons might be systemic. Hence, it encourages for the further investigation of sensory nerve system activity in tumor-bearing hosts.

Up-regulated expression of chemokines in tumor-treated DRG neurons has been confirmed in functional assays revealing that melanoma-treated DRG cells are the stronger chemoattractants for MDSC than control neurons. MDSC are represented by a mixed population of immature myeloid cells with strong immunosuppressive activities [38]. In cancer, increased levels of MDSC have been reported both systemically and within the tumor microenvironment and their protumorigenic role has been well documented [19, 39]. Thus, our *in vitro* results unveil the tumor-activated sensory neurons to support the attraction of MDSC to the tumor site, which, in turn, favors the development of the immunosuppressive tumor microenvironment. Moreover, our data *in vivo* demonstrate the presence of DRG neurons to provide higher levels of granulocytic MDSC in melanoma (Fig 6), which also associates with tumor growth acceleration *in vivo* (Fig 2). Interestingly, a preferential expansion of a granulocytic subset of MDSC has been demonstrated in almost all tested murine tumor models [40]. Patients with renal, pancreatic, colon and lung cancers display elevated levels of granulocytic MDSC [41–44], while elevated MDSC levels are connected with cancer progression and tumor-induced immune dysfunction [19].

In summary, our data provide the first proof-of-concept for melanoma-activated sensory neurons to contribute to tumor growth by supporting the development of the local protumorigenic microenvironment. Of a higher importance is the notion that in the absence of initial inflammation this formed tumor-neuronal-immune axis results in the formation of the protumorigenic immunological microenvironment. Further evaluation of the complex multidirectional interactions between the tumor, somatosensory nervous system and immune system along with identification of pathophysiological pathways involved in these communications should provide novel insights into the primary and metastatic tumorigenic mechanisms.
